# Identification of cellular pathways affected by Sortin2, a synthetic compound that affects protein targeting to the vacuole in *Saccharomyces cerevisiae*

**DOI:** 10.1186/1472-6769-8-1

**Published:** 2008-01-07

**Authors:** Lorena Norambuena, Jan Zouhar, Glenn R Hicks, Natasha V Raikhel

**Affiliations:** 1 Department of Biology, Faculty of Sciences, University of Chile, Santiago, Chile; 2 Departamento de Genética Molecular de Plantas, Centro Nacional de Biotecnología, Consejo Superior de Investigaciones Científicas, E-28049 Madrid, Spain; 3 Center for Plant Cell Biology and Department of Botany and Plant Sciences at University of California. 2109 Batchelor Hall, University of California Riverside, CA 92521 USA

## Abstract

**Background:**

Sortin2 is a low mass compound that interferes with vacuolar delivery of proteins in plants and yeast. The Sortin2 phenotype was tested in *Arabidopsis thaliana *and found to be reversible upon drug removal, demonstrating the ability of chemical genomics to induce reversible phenotypes that would be difficult to achieve using conventional genetics [1]. However, standard genetic methods can be used to identify drug target pathways in a high-throughput manner.

**Results:**

In this study, we analyzed structure-function relationships of Sortin2 using structural analogues. The results show the key roles of sulphite substitution and a benzoic acid group. A Sortin 2 hypersensitivity screen for the induced secretion of a vacuolar cargo protein was done utilizing a yeast haploid deletion library. Using bioinformatics approaches, we highlighted functional information about the cellular pathways affected by drug treatment which included protein sorting and other endomembrane system-related processes.

**Conclusion:**

Chemical, genomic and genetics approaches were used to understand the mode of action of Sortin2, a bioactive chemical that affects the delivery of a vacuolar protein. Critical features of Sortin2 structure necessary for bioactivity suggest a binding pocket that may recognize two ends of Sortin2. The genome-wide screen shows that Sortin2 treatment in yeast affects primarily components within the endomembrane system. This approach allowed us to assign putative functions in protein sorting for fifteen genes of previously unknown function.

## Background

Endomembrane trafficking in eukaryotes is essential for the intracellular delivery of cargoes and membranes. As the site of protein degradation, nutrient recycling and storage of biological components, the vacuole is one of the key compartments of the endomembrane system. In addition, the mechanisms implicated in the delivery of cargoes to the vacuole have been delineated mainly by yeast genetic screens. A relatively new approach for discovering cellular pathways that takes advantage of yeast genetics is chemical genomics, which uses small bioactive molecules that can affect biological pathways [2,3]. The physiological effects of such drugs may be appreciated and exploited long before the corresponding targets are identified. However, the eventual identification of target proteins within drug-sensitive pathways is necessary to discover and characterize intracellular networks [4-10].

Using forward chemical genomics, we identified fourteen low mass compounds that interfere with the delivery of the vacuolar resident protein carboxypeptidase Y (CPY) in *Saccharomyces cerevisiae *[1]. These compounds are able to trigger the secretion of CPY, mimicking the *vacuole protein sorting *(*vps*) phenotype [11]. Two of these compounds, Sortin1 and Sortin2, result in the secretion of CPY in *Arabidopsis thaliana *[1]. To understand Sortin2 activity in more detail, we focused on two objectives: to understand the structural features of Sortin2 necessary for its activity and to analyze *S. cerevisiae *cellular pathways affected by the chemical. The bioactivity of different Sortin2 analogues highlighted structural domains important for Sortin2 activity. In order to identify the cellular pathway(s) affected by Sortin2, a genetic screen was performed for strains hypersensitive to this chemical in a *S. cerevisiae *haploid deletion collection. Among 4,800 clones screened, 217 strains were hypersensitive to Sortin2. Bioinformatics analysis indicated that the Sortin2 dataset was enriched in components related to protein trafficking and localized primarily to the endomembrane system, particularly endosomes. Our approach permitted an initial functional assignment of fifteen genes of previously unknown function to protein trafficking pathways.

## Results

**Figure 1:**
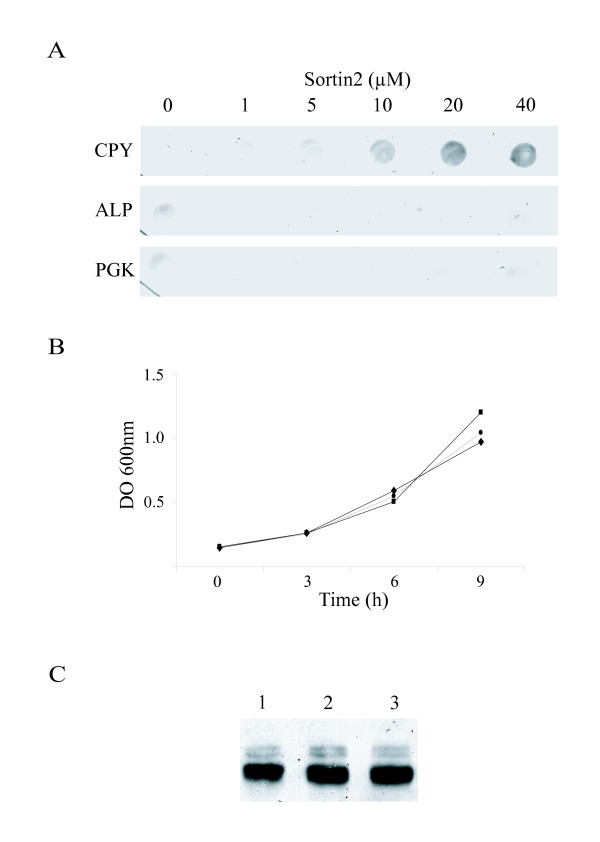
Effects of Sortin2 treatments on *S. cerevisiae*. (A) Dot-blot secretion assay using monoclonal antibodies raised against CPY, alkaline phosphatase (ALP) and 3-phosphoglycerate kinase (PGK). (B) Yeast growth in presence of 1% DMSO (●), 2 μg/ml (4.7 μM) Sortin2 (◆) and 20 μg/ml (47 μM) Sortin2 (■). (C) Western blot using CPY antibody of cell fractions of yeast upon 1% DMSO (1), 2 μg/ml (4.7 μM) Sortin2 (2) and 20 μg/ml (47 μM) Sortin2 (3) treatment.

Carboxypeptidase Y (CPY) is a soluble vacuolar hydrolase that is sorted from the late Golgi to the late endosome and subsequently trafficked to the vacuole [12-14]. In yeast, Sortin2 interfered with CPY sorting resulting in secretion to the media [1]. Sortin2 also phenocopied *vps *mutants without inhibiting yeast growth, at concentrations ranging from 4.7 μM to 47 μM (Fig 1a and 1b). Upon Sortin2 treatment, there was no detectable presence in the medium of the proteins such as alkaline phosphatase (ALP), a membrane-bound vacuolar protein, or 3-phosphoglycerate kinase, a cytoplasmic protein (Fig. 1a). These controls indicated that CPY in the medium of Sortin2-treated cells was not due to cell lysis. The total amount of expressed CPY protein was not significantly affected by Sortin2 treatment indicating that its secretion was due to an alteration in cargo delivery rather than simple overloading of endomembrane compartments (Fig 1c). Therefore, we concluded that Sortin2 targeted components involved in the delivery of CPY from the endoplasmic reticulum to the vacuole.

### Sortin2 structure-bioactivity relationships

**Figure 2:**
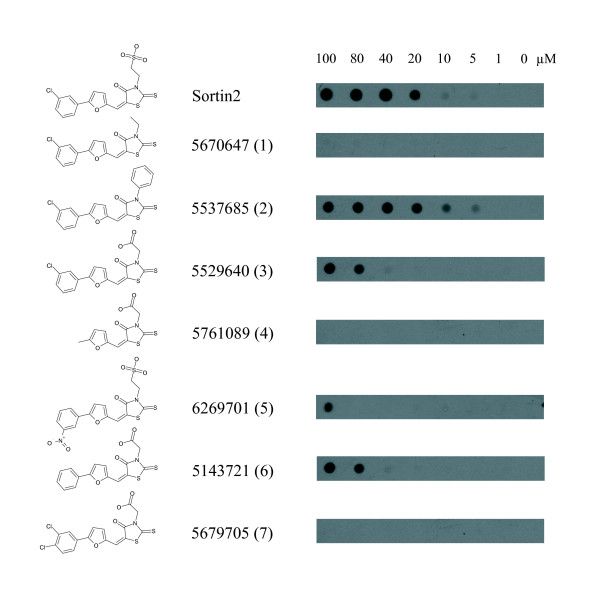
Structure-activity relationship of Sortin2 structural analogs. The ability to trigger secretion of CPY was tested for all compounds shown. Compounds are referred by the identification numbers assigned by manufacturer.

In order to understand the structural determinants of Sortin 2 necessary for bioactivity, a panel of chemicals that were similar in structure to Sortin2 were examined. The Sortin2 contains discrete domains: a chlorobenzene, a furan, a thiazolidine ring, and a sulphite group. The activity of structural analogs as promoters of CPY secretion is shown in Figure 2. For convenience, the potency of Sortin 2 and its analogues was defined as the minimum concentration at which activity was observed.

Chemical **1 **(Fig. 2) represented the Sortin2 structure minus the sulphite group. This analogue was inactive indicating that the sulphite group was critical for binding of Sortin2 to its target. Chemical **2 **which had a benzoic ring substitution instead of the sulphite group had the same activity as Sortin2. Chemical **3 **had a carboxyl group instead of the sulphite group. It was bioactive; however, it had a lower potency (40 μM) than Sortin2 and chemical **2 **(5 μM). Taken together, these data suggest that the interaction between Sortin2 and its target probably required a dense electron cloud in order to affect target activity and function. This could be direct or indirect by affecting the overall conformation of Sortin2.

Chemical **4 **lacked the chlorobenzene ring, but did not alter the delivery of CPY to the vacuole. We further tested the importance of the chlorine on the benzene ring by testing another chemical **(5) **which had a nitro group substitution. Chemical **5 **was twenty times less active than Sortin2 which indicated that the presence of the chloride was important for full activity. However, chemical **6**, in which the chloride was absent showed a potency that was similar to that of chemical **5 **supporting the notion that the halogen atom was not a key feature for bioactivity. Chemical **7 **which had two chlorides associated with the benzoic group was completely inactive, perhaps due to steric effects. Steric hindrance may also explain the low activity of chemical **5 **since the major difference was the chloride and nitro groups. Overall we concluded that the sulphite group and the benzoic acid ring were essential for Sortin2 bioactivity.

### Screening for yeast mutants hypersensitive to Sortin2

**Figure 3:**
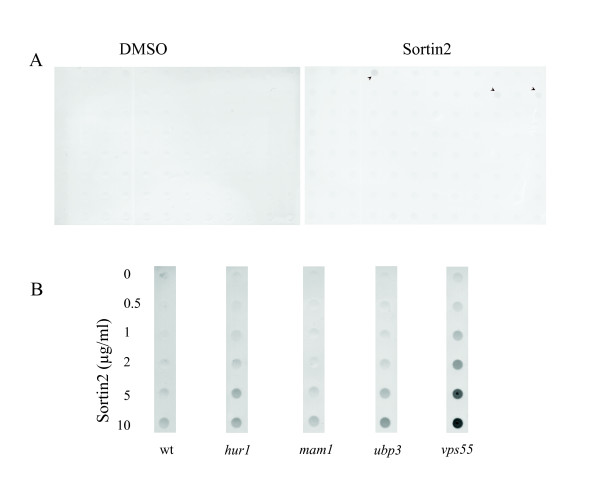
Identification of Sortin2 hypersensitive mutants. (A) Primary screen: 4827 haploid deletion strains were challenged with 1% DMSO (DMSO) or Sortin2 at 2 μg/ml (4.7 μM) (Sortin2). Culture media was analyzed for the presence of CPY by dot blot assay. Clones secreting significant amounts of CPY compared to the negative control were selected as putative mutants. The arrowheads show the primary hits. (B) Secondary screen: 243 putative mutants were tested on DMSO and varying concentrations of Sortin2. Threshold was defined as the concentration of Sortin2 that had no residual CPY secretion on a wild type parental line (wt, 2 μg/ml). For CPY secretor strains, the strain was considered as hypersensitive to Sortin2 if the CPY secretion in the presence of Sortin2 was in excess of that displayed in the presence of DMSO. Different scenarios are shown as examples of the secondary screen results: a false positive strain (*mam1*), a Sortin2 hypersensitive strain (*ubp3*), and two Sortin2 hypersensitive strains secreting CPY on control conditions (*hur1 *and *vps55*).

In order to identify cellular pathways affected by Sortin2 a haploid yeast deletion library was screened for mutants that secreted CPY at concentrations of Sortin2 that did not trigger detectable CPY secretion in the wild type haploid strain. Of the collection of 4,800 strains, 243 putative hypersensitive mutants were identified in the primary screen (Fig. 3a). Each putative mutant was subsequently exposed to various concentrations of Sortin2 to verify the drug dependency of their secretion phenotype (Fig. 3b). A mutant was considered verified as hypersensitive if significant CPY secretion was detected at a concentration of Sortin2 less than that necessary to result in CPY secretion from the parental line (Fig. 3b). Ninety percent (217) of the strains were verified as hypersensitive to Sortin 2. The large number of successfully verified strains demonstrated the robustness of the primary screen.

### *Vacuolar protein sorting *(*vps*) mutants and hypersensitivity to Sortin2

Sixty-one yeast deletion strains were known previously to have impaired sorting of CPY; these are referred to as *vps *mutants. Forty-one *vps *mutants were identified as drug-dependent in our screen with respect to Sortin2. These included mutants in all six classes of *VPS *genes (Additional file 1). Class E is involved in protein sorting to lumenal vesicles of the MVB (multi-vesicular body). This family contains 16 members plus *VTA1 *and *HSE1*; however, the latter two genes were not described as *VPS *genes [11]. Remarkably, fourteen out of sixteen members (88.9%) of the *VPS *Class E were hypersensitive to Sortin2, the class with highest representation among *VPS *genes. Interestingly, the remaining 20 *vps *mutants displayed no secretion of CPY when treated with Sortin2. The screen, thus, discriminated between deletion and Sortin2-induced *vps *phenotypes. This indicated that the Sortin2 phenotype was specific and distinct, rather than a broad effect on secretion, when compared to the majority of the *vps *mutants. As part of this screen, we analyzed the drug sensitivity of 148 strains identified previously in a screen for mutants that secreted CPY [15]. Among these mutants, 91 were hypersensitive to Sortin2 when compared to the untreated control. The absence of a hypersensitive response from the other 57 mutants indicated that Sortin2 was selective for specific elements of the vacuole targeting machinery.

### Analyses of identified ORFs

FunCat [16] was used to classify the Sortin2 hypersensitive dataset into functional categories relative to the Saccharomyces genome (Table 1, Additional file 2). Some categories showed statistically significant over-representation such as "interaction with the environment" and "biogenesis of cellular components" which were enriched 1.7- and 1.5-fold, respectively (p ≤ 0.04) (Table 1). The greatest enrichment were the categories "protein fate" and "cellular transport, transport facilities and transport routes" which displayed 2.5- and 3-fold enrichment, respectively (p = 1.51 E-19 and 2.99 E-29; Table 1). In terms of subcellular location, the greatest over-representation corresponded to the category "endosomes" with a 17.5-fold enrichment compared to the yeast genome (p = 6.2 E-35) (Table 1). The "Golgi apparatus", "transport vesicles", "vacuole" and "punctuate composite" categories were enriched between 3.5- and 5.4-fold with statistically significant over-representation as shown by the p-values in Table 1. These results revealed that processes within the endomembrane system are strongly and specifically affected by Sortin2 treatment.

Forty-seven percent of the Sortin2 dataset was associated with the GO term "Transport". The set was analyzed by Term Finder tools, and significantly, the sole descendant terms included "Protein Transport", "Intracellular Transport", "Vesicle-mediated Transport" and "Secretory Pathway" highlighting the involvement of the majority of identified ORFs in protein trafficking pathways (data no shown). We also applied the Term Finder tool to analyze the association of the Sortin2 dataset with granular GO terms that were linked to a cellular component (Additional file 3). Within the Sortin2 hypersensitive dataset, we identified members of crucial Golgi and endosomal sorting complexes, their interacting partners and other resident proteins associated all directly in CPY sorting (Additional file 3). In the endosomal compartment, which is the destination for CPY after exiting the late Golgi, we identified members of the three known endosomal and the MVB sorting complexes. Systematic deletion of ESCRT (Endosomal Sorting Complex Required for Transport) genes was reported to cause reduced telomere length [17]. Interestingly, we identified ESCRT-unrelated telomere length mutants as hypersensitive in our screen. In addition several mutants linked to chromatin remodelling were identified (Additional file 3). Overall, we concluded that the Sortin2 hypersensitive ORFs were located preferentially within the endomembrane system and included many molecular components related to the CPY delivery. Our screening results also supported the hypothesis previously proposed that interactivity occurs between the endomembrane system, chromatin remodelling, and telomere maintenance processes.

Importantly, the sublethal conditions of the screening assay yielded an unbiased set of mutants. The dataset did not include any ABC transporters or ergosterol biosynthetic genes which are related to multi-drug resistance [18] suggesting that the screen distinguished between induced *vps *phenotypes and general drug sensitivity.

**Table 1:** Functional and locational gene product categorization of Sortin2 hypersensitive ORFs.

	Representation on dataset			Enrichment on dataset (-fold)
	Sortin2	Genome	p-value	
**FUNCTIONAL CATEGORY**				

Cellular transport, transport facilities and transport routes	50.7	16.9	2.99 E-29	3.0
Protein fate (folding, modification, destination)	46.2	18.8	1.51 E-19	2.5
Biogenesis of cellular components	21.3	14	0.003	1.5
Interaction with the environment	12.9	7.55	0.004	1.7

**LOCATIONAL CATEGORY**				

Endosome	16.4	0.94	6.2 E-35	17.5
Golgi	13.9	2.57	1.0 E-13	5.4
Transport vesicles	11.4	2.29	1.1 E-10	5.0
Punctate composite	8.95	2.29	6.3 E-07	3.9
Vacuole	16.4	4.63	1.1 E-10	3.5
ER	15.9	9.1	0.0011	1.8

Category representation of the abundance within Sortin2 hypersensitive (201 genes) and the Saccharomyces genome (6131 genes). P-value was calculated using hypergeometric distribution as statistical test. P-values < 0.0005 were considered as statistical significant.

### Unknown genes

Of 217 Sortin2 hypersensitive mutants, 30 were classified as unknown according to the SGD databases. Among these ORFs, several were dubious genes with known genes on the complementary DNA strand. It is also possible that the Sortin2 phenotype resulted from a defect in an authentic gene whose ORF overlapped the dubious ORF. These dubious genes are listed with their corresponding known ORFs in parenthesis in Table 2. *YIP4 *encodes a protein that mediates vesicular trafficking via its interaction with Rab GTPases [19] supporting the dubious character of *YGL199C*. Similarly, Gim5p is a member of a protein complex that promotes formation of functional α- and γ-tubulin [20]. This complex includes Gim4p that was identified in our screen; thus *YML094C-A *is probably a dubious ORF.

**Table 2:** Unknown function ORF of the corresponding deletion strains identified as Sortin2 hypersensitive mutants.

**Unknown genes categories**	**ORF**
Dubious	*YGR064W (SPT4)*
	*YDL096C (PMT1)*
	*YNR005C (VPS27)*
	*YOR331C (VMA4)*
	*YPR050C (MAK3)*
	*YDR455C (NHX1)*
	*YML012C-A (SEL1)*
	*YKR035C (DID2)*
	*YGL199C (YIP4)*
	*YML094C-A (GIM5)*
	*YOL079W (REX4)*

CPY secretor deletion strain	*YLR426W*
	*YNL080C*
	*YDR525W-A/SNA2*
	*YDR105C/TMS1*

Not previously associated to sorting pathway	*YCL075W*
	*YCR095C*
	*YDR357C*
	*YGL079W*
	*YIL039W*
	*YIL041W/Gvp36p*
	*YIL130W/ASG1*
	*YJL077C/ICS3*
	*YKR088C/TVP38*
	*YLL049W*
	*YLR361C/DCR2*
	*YMR158C-B*
	*YMR315W*
	*YNL063W/MTQ1*
	*YOL111C*

The name in parenthesis correspond to the known gene on the complementary DNA strain of dubious genes

Other unknown genes were found in high-throughput screens that focused on genetic/physical interactions and protein localization [21-23]. This annotation supports putative roles for the corresponding gene products in protein trafficking. Ygr206wp, for example, was identified as endosomal and more recently as a component of the ESCRT-1 complex [23-25]. YGR206wp and Ylr426wp physically interact with the V-ATPase subunit Vma6p [21]. Their corresponding genes together with *YNL080C*, *SNA2 *and *TMS1 *were the only uncharacterized ORFs from our dataset that overlapped previously unknown genes whose deletion resulted in CPY secretion [15]. Excluding the 10 dubious ORFs described above, the remaining 15 unknown genes have not been associated previously with protein sorting pathways. Among these unknowns, Yil041wp (Gvp36p) was localized to Golgi vesicles [26]. The mutants for the unknown ORFs *YDR357C *and *YGL079W *were hypersensitive to Sortin2 and their gene products were identified as interacting partners in a two-hybrid assay [22]. The GFP fusion of Ygl079wp co-localizes with an endosome marker [23]. In addition, Ydr357cp and Yll049wp, both from the Sortin2 dataset, physically interact with the autophagy protein Atg17p [21] suggesting a role in the endomembrane system. Similarly, *YIL039W *and *YLR361C (DCR2) *genetically interact with the Golgi residents Ric1p and Ypt6p [27], both of which mediate vesicular trafficking at the Golgi apparatus. Tvp38p (Figure 3b) is an integral membrane protein localized to late Golgi vesicles along with the v-SNARE Tlg2p [26].

To test the interaction of mutants in unknown ORFs with Sortin2, active and inactive analogues were tested for their ability of trigger CPY secretion in the corresponding deletion mutants for *YDR525W-A/SNA2, YDR105C/TMS1*, *YGL079W, YLL049W, YKR088C/TVP38 *(data no shown). All of the mutants were more hypersensitive compared to their parental strain when grown in the presence of bioactive compounds **2 **and **5**. In contrast, none of the strains tested were hypersensitive to the inactive Sortin2 analogues **1**, **4 **and **7**. Therefore, the Sortin2 hypersensitivity of mutants in these genes of unknown function was specific for analogues possessing features important for Sortin2 bioactivity. There were no detectable ALP or PGK proteins in the culture media for any Sortin2 analogs (data no shown) indicating that the CPY secretion was not due to the cell lysis.

In summary, we assigned a putative role in protein trafficking to 15 ORFs of unknown function in *S. cerevisiae *based on their Sortin2-induced *vps *phenotypes. The actual role of these ORFs will be addressed in future studies.

## Discussion

In this study, we analyzed the structure-activity relationships and the cellular pathways affected by Sortin2 which triggers the secretion of the soluble vacuolar protein CPY in *S. cerevisiae *and *A. thaliana*. Our Sortin2 hypersensitive screen identified genes whose loss of function resulted in no vacuolar sorting defects; however, a sorting defect occurred when these mutants were challenged with the drug.  Furthermore, our analysis implicated a number of genes of unknown function in the process of vacuolar protein sorting. Therefore, this approach could be of general use for discovering the functional identity of uncharacterized genes.

The use and analysis of Sortin2 structural analogs allowed us to define the sulphite substitution and benzoic acid group on Sortin2 as key determinants for bioactivity and perhaps target binding. Interaction with a target would probably require both ends of the molecule suggesting a binding pocket that recognizes one face of Sortin2. However, the possibility of multiple binding pockets or targets can not rule out. It is possible to speculate that the dimensions of the binding pocket in the cognate target are very exacting for activity resulting in lower potency or no activity as observed for several Sortin2 analogs. The compounds tested are within the parameters set by Lipinski [28] indicating that compound permeability would not be an issue.

We screened for enhanced secretion in the presence of Sortin2 using a haploid deletion library. If the drug has a single target, deletion of the gene encoding the target would result in a strain whose phenotype could not be enhanced further by the drug. Thus, our approach would not identify a cognate target. However, we anticipated that this hypersensitive screen would identify proteins that were either in the same pathway or in a pathway connected to the Sortin2 target. The main pathway affected by Sortin2 is the endomembrane system although the effect could be indirect. The intracellular location displaying the greatest enrichment was the endosomes suggesting that the Sortin2 target may be affecting mainly the endosomal compartments which precede the final localization of CPY. Consistent with this idea, almost 90% of the *vps *Class E mutants, which have an aberrant MVB (the "Class E compartment"), were hypersensitive to Sortin2. Sortin2 is able to mimic the *vps *phenotype; however, its effect on MVB structure has not been tested yet.

Sortin2 will be useful for probing conserved processes such as endomembrane trafficking in other organisms, and the powerful genomics available will permit the identification of cognate targets.

## Conclusion

In this study, we combined chemical, genomic and genetics approaches to understand the mode of action of Sortin2, a bioactive chemical that affects the delivery of a vacuolar protein. Several features of Sortin2 structure are critical for bioactivity suggesting a binding pocket that recognizes one face of the molecule.

The genome-wide screen in yeast showed that Sortin2 mainly affected components within the endomembrane system. Other cellular functions affected by Sortin2 may highlight interactions between cellular processes. Our approach allowed us to assign a putative function in protein sorting for 15 genes of unknown function that were not associated previously with protein trafficking pathways.

## Methods

### Protein secretion assay

The BY4742 haploid parental line (MATalpha *his3Δ1 leu2Δ0 lys2Δ0 ura3Δ0*) was grown in yeast extract/peptone/dextrose (YPD) medium supplemented with the designated compounds or 1% DMSO as a negative control. Cultures were grown for 72 hours at 30°C. Growth medium was analyzed using monoclonal antibodies against CPY, ALP and 3-phosphoglycerate kinase (Molecular Probes) by dot-blot as described previously [1].

### Sortin2 analogs and substructures

Sortin2 and its analogues and substructures were obtained from Chembridge (San Diego, CA) and referred to by the identification numbers assigned by the manufacturer. Analogue and substructure searches were done using the Hit2Lead database (Chembridge), SciFinder database, and the ChemMine comparative chemical database [29,30].

### Sortin2 Hypersensitivity Screening

The *S. cerevisiae *haploid deletion library was generated using the BY4742 parental strain containing 4,800 yeast clones (Open Biosystems, Huntsville, AL). The primary screen utilized the microplate format of the deletion library. The library was grown on 2 mg/l (4.7 μM) Sortin2 and 1% DMSO. Growth medium was analyzed for secreted CPY as described previously [1]. The mutant strains identified in the primary screen were further analyzed for CPY secretion at various concentrations of Sortin2 (0.5, 1, 2, 5, and 10 mg/l) in a verification screen. Mutants were considered verified as hypersensitive if significant CPY secretion was detected at 2 mg/l of Sortin2.

### Bioinformatics analyses

The Functional Categorization (FunCat) and Cellular Location (CellLoc) of the identified genes were acquired form The **M**unich **I**nformation Center for **P**roteins **S**equences [31]. To retrieve information about each deleted ORF, we queried the Saccharomyces Genome Database (SGD, [32]). The SGD tools were also used to assign the granular or general Gene Ontology (GO) terms to identified ORFs, using the GO Term Finder or the GO Slim Mapper, respectively.

## Abbreviations

Carboxypeptidase Y (CPY)

Vacuole protein sorting (vps)

Alkaline phosphatase (ALP)

Open reading frame (ORF)

Multi vesicular body (MVB)

Gene Ontology (GO)

Endosomal Sorting Complex Required for Transport (ESCRT)

## Authors' contributions

LN tested Sortin2 specificity in terms of secretion and growth, performed the structure-bioactivity analysis, conducted the screening, analyzed and organized the bioinformatics data, designed this study and wrote this manuscript. JZ designed and carried out the screening, analyzed the bioinformatics data and drafted the manuscript. GH made substantial intellectual contributions to generate this manuscript and edited the manuscript. NR conceived the study and participated in its design and coordination. All authors read and approved the final manuscript.

## Supplementary Material

Additional File 1 Supplemental Table. *VPS *genes and hypersensitivity to Sortin2. List of VPS gene identified as hypersensitive to Sortin2. A table. Click here for file

Additional File 2 Supplemental Table 2. Sortin2 hypersensitive mutant dataset was analyzed by FunCat. A table. Click here for file

Additional File 3 Term Finder analysis of gene association with granular GO Component terms. Analysis of datasets using Term Finder tool. A table. Click here for file
