# Supplementary material for: Identification of cellular pathways affected by Sortin2, a synthetic compound that affects protein targeting to the vacuole in Saccharomyces cerevisiae
Source: BMC Chem Biol. 2008 Jan 7;8:1. doi: 10.1186/1472-6769-8-1 (PMC2265672; doi:10.1186/1472-6769-8-1)
Supplement: Additional File 1 — Supplemental Table. VPS genes and hypersensitivity to Sortin2. List of VPS gene identified as hypersensitive to Sortin2. A table. [file 1472-6769-8-1-S1.pdf]

Supplemental Table 1: *VPS* genes and hypersensitivity to Sortin2

vps mutant class	Sortin2 hypersensitive genes
**Class A** (11)	VPS8 VPS13 VPS29 VPS38 VPS55 VPS63
**Class B** (17)	VPS5 VPS17 VAM6/VPS39 VPS41 VAM7/VPS43 VPS51 VPS54 VPS61 VPS66 VPS71 VPS72
**Class C** (4)	VPS16
**Class D** (9)	VPS3 VPS9 VPS15 PEP7/VPS19 VPS34 VPS45
**Class E** (16)	VPS4 VPS20 SNF8/VPS22 STP22/VPS23 VPS24 VPS25 VPS27 VPS28 BRO1/VPS31 SNF7/VPS32 VPS36 SRN2/VPS37 NHX1/VPS44 DID2/VPS46
**Class F** (6)	VPS1 VPS68 VPS71

List of VPS gene identified as hypersensitive to Sortin2. The number of class members is indicated on parenthesis.
