# Supplementary material for: Identification of cellular pathways affected by Sortin2, a synthetic compound that affects protein targeting to the vacuole in Saccharomyces cerevisiae
Source: BMC Chem Biol. 2008 Jan 7;8:1. doi: 10.1186/1472-6769-8-1 (PMC2265672; doi:10.1186/1472-6769-8-1)
Supplement: Additional File 2 — Supplemental Table 2. Sortin2 hypersensitive mutant dataset was analyzed by FunCat. A table. [file 1472-6769-8-1-S2.pdf]

Supplemental Table 2: Gene functional categorization of genes deletions identified as Sortin2 hypersensitive.

FUNCTIONAL CATEGORY	Gene set
02 energy	*YEL053c YHR060w YJR033c YMR164c YOL079w*
10 cell cycle and dna processing	*YAL011w YBR245c YCR033w YCR094w YDL074c YDL117w YDL185w YDR069c YDR137w YDR295c YDR310c YDR317w YDR334w YDR359c YDR469w YDR485c YER111c YER123w YGL173c YGR063c YHR004c YHR115c YHR191c YIL153w YJR060w YKL113c YKR019c YLR015w YLR085c YLR361c YLR399c YML041c YNL021w YNL064c YNL082w YNL098c YNL107w YOL076w YPL055c YPL241c YPR179c*
11 transcription	*YBR240c YBR245c YCR094w YDL005c YDR295c YDR310c YDR334w YDR443c YER111c YGL070c YGL173c YGR063c YHR178w YIL130w YJR060w YJR102c YKR019c YLR015w YLR039c YLR399c YLR417w YMR164c YNL021w YNL107w YNL136w YNR015w YPL002c YPR179c*
12 protein synthesis	*YJL136c YLR185w YMR142c YNL067w YOL033w*
14 protein fate (folding, modification, destination)	*YAL002w YAL014c YBL082c YBR097w YBR131w YBR164c YCL008c YCR033w YDL074c YDL077c YDL090c YDL095w YDL185w YDL192w YDR069c YDR080w YDR140w YDR295c YDR323c YDR425w YDR469w YDR485c YDR495c YEL003w YEL013w YEL053c YER123w YFR019w YFR021w YGL038c YGL095c YGR036c YGR105w YGR270w YHL002w YHL031c YHR012w YHR060w YJL036w YJL184w YJR102c YJR117w YJR125c YKL041w YKL080w YKL119c YKL197c YKR001c YKR020w YKR035w-a YLL040c YLR015w YLR025w YLR085c YLR119w YLR240w YLR309c YLR360w YLR417w YLR447c YML013w YML041c YML097c YMR004w YMR274c YNL021w YNL059c YNL064c YNL136w YNL315c YNR006w YOL018c YOL076w YOL111c YOL129w YOR069w YOR106w YOR132w YOR219c YOR270c YOR357c YPL002c YPL045w YPL051w YPL055c YPL065w YPL084w YPL125w YPL172c YPR051w YPR139c YPR173c YPR179c*

16 protein with binding function or cofactor requirement (structural or catalytic)	*YBR245c YCL008c YDL074c YEL003w YEL013w YFR021w YGL070c YGL095c YGR270w YHL002w YHR060w YJL036w YJR060w YJR117w YJR125c YKL197c YKR019c YKR020w YNL079c YNL082w YNL315c YNR006w YOR357c YPR173c*
18 regulation of metabolism and protein function	*YBR131w YDL077c YDR080w YDR137w YIL044c YIL153w YKR019c YLR039c YLR386w YLR417w YML097c YNL064c YOR070c*
20 cellular transport, transport facilities and transport routes	*YAL002w YAL014c YBR021w YBR097w YBR106w YBR127c YBR131w YBR164c YBR288c YCL008c YCR053w YDL185w YDL192w YDR027c YDR069c YDR080w YDR137w YDR323c YDR425w YDR456w YDR485c YDR495c YEL013w YEL031w YEL051w YFR019w YFR021w YGL005c YGL095c YGL148w YGL167c YGL212w YGL223c YGR260w YGR261c YHL002w YHL031c YHL040c YHR012w YHR026w YIL044c YJL004c YJL024c YJL036w YJL117w YJR040w YJR044c YJR102c YJR106w YJR125c YKL041w YKL080w YKR001c YKR020w YKR035w-a YLL040c YLR025w YLR039c YLR085c YLR119w YLR181c YLR240w YLR262c YLR268w YLR309c YLR360w YLR370c YLR417w YLR447c YML001w YML013w YML041c YML067c YML071c YML097c YMR004w YMR077c YNL041c YNL051w YNL059c YNL064c YNL079c YNR006w YOL018c YOL129w YOR069w YOR070c YOR106w YOR132w YOR216c YOR270c YPL002c YPL045w YPL051w YPL065w YPL084w YPL125w YPL195w YPR032w YPR079w YPR139c YPR173c*
30 cellular communication/signal transduction mechanism	*YDL090c YKR019c YLR240w YNL098c YPL084w YPR079w*
32 cell rescue, defense and virulence	*YBR006w YBR131w YDR456w YFR019w YGL095c YHL040c YHR060w YIL153w YJL184w YKL113c YNL064c YNL098c YNL315c YPL084w*
34 interaction with the environment	*YBR127c YCL058c YDL185w YDR069c YDR202c YDR456w YEL031w YEL051w YGL095c YGL167c YHR026w YHR060w YHR178w YJL117w YJR033c YJR040w YJR117w YKL080w YKL119c YLR110c YLR447c YNL079c YNL098c YOR219c YOR270c YPL084w*
40 cell fate	*YDR137w YGL095c YKL113c YNL021w YNL079c YNL098c YPL055c*

42 biogenesis of cellular components	*YAL011w YBR131w YBR168w YDL074c YDL077c YDR027c YDR069c YDR080w YDR202c YDR469w YEL003w YEL013w YFR019w YFR021w YGL095c YGL212w YGR036c YHR004c YHR012w YJL004c YJL184w YJR060w YJR106w YJR125c YKL197c YKR001c YKR019c YKR020w YLR015w YLR110c YLR309c YLR370c YNL079c YNL136w YNL315c YOL018c YOL076w YOR068c YPL045w YPL055c YPL241c YPR032w YPR173c*
43 cell type differentiation	*YCR094w YDR069c YFR021w YGL095c YGL173c YHR004c YJR125c YLR025w YLR399c YMR164c YNL079c YNL098c YPR032w*
99 unclassified proteins	*YCR095c YDR105c YDR357c YDR525w-a YEL044w YGL079w YGR206w YIL039w YIL041w YJL077c YJR118c YKR088c YLR426w YMR315w YNL080c*

Sortin2 hypersensitive mutant dataset was analyzed by FunCat.
