# Supplementary material for: Identification of cellular pathways affected by Sortin2, a synthetic compound that affects protein targeting to the vacuole in Saccharomyces cerevisiae
Source: BMC Chem Biol. 2008 Jan 7;8:1. doi: 10.1186/1472-6769-8-1 (PMC2265672; doi:10.1186/1472-6769-8-1)
Supplement: Additional File 3 — Term Finder analysis of gene association with granular GO Component terms. Analysis of datasets using Term Finder tool. A table. [file 1472-6769-8-1-S3.pdf]

Supplemental Table 3: TermFinder analysis of gene association with granular GO:component terms.

GO ID #	GO: component	Sortin2 dataset
5794	Golgi apparatus	ARF1 ARL1 ARL3 COG1 COG5 COG6 COG7 COG8 ERV41 GEF1 GYP1 IMH1 OCH1 PMR1 RIC1 RGP1 RUD3 SEC22 STE13 SYS1 TLG2 YPT6 VPS15 VPS45 VPS51 VPS54
17119	Golgi transport complex	COG1 COG5 COG6 COG7 COG8
93	GARP complex	VPS51 VPS54
5768	endosome	ARN1 BRO1 CCZ1 CDC50 DID2 GEF1 HSE1 MRL1 NHX1 PEP7 SNF7 SNF8 SNX41 SRN2 STP22 SYN8 TLG2 VPS5 VPS13 VPS17 VPS20 VPS24 VPS25 VPS27 VPS28 VPS29 VPS36 VPS41 VPS55 VTA1 YGL079W YGR206W
813	ESCRT I complex	VPS37 VPS23 VPS28
814	ESCRT II complex	VPS22 VPS25 VPS36
815	ESCRT III complex	VPS32 VPS20 VPS24
42175	nuclear envelope-ER network	ARL3 CAX4 ERV41 NEM1 PMT1 RCE1 SPF1 STE24 VMA21 VMA22 VPH2
16585	chromatin remodeling complex	ARP5 ARP6 BRE2 ISW1 SDC1 SWC3 SWR1 VPS71 VPS72 YAF9
812	SWR1 complex	ARP6 SWC3 SWR1 VPS71 VPS72 YAF9
5770	late endosome	CCZ1 CDC50 DID2 MRL1 NHX1 VPS55 VTA1
5773	vacuole	ATG18 CCZ1 FAB1 TFP1 TMS1 VAC8 VAC14 VAM3 VAM6 VAM7 VAM10 VMA2 VMA5 VMA6 VMA8 VMA9 VMA16 VPH1 VPS16 VPS41 VPS68 YCK3 YPT7
16471	hydrogen-translocating V-type ATPase complex	TFP1 VMA2 VMA5 VMA6 VMA8 VMA9 VMA16 VPH1
43291	RAVE complex	RAV1 RAV2
30897	HOPS complex	VAM6 VPS16 VPS41

Analysis of datasets using Term Finder tool.
